# Low seroprotection rate for meningococcus serogroup C in the adult HIV-1-infected population in Austria

**DOI:** 10.1007/s00508-019-01561-4

**Published:** 2019-10-24

**Authors:** Katharina Grabmeier-Pfistershammer, Kay Holleis, Sandra Rosskopf, Peter Steinberger, Veronique Touzeau-Roemer, Wolfgang Poeppl, Armin Rieger

**Affiliations:** 1 Institute of Immunology, Center for Pathophysiology, Infectiology and Immunology, Medical University Vienna, Vienna, Austria; 2 Department of Dermatology, Medical University Vienna, Vienna, Austria; 3 Department of Dermatology and Tropical Medicine, Military Medical Cluster East, Austrian Armed Forces, Vienna, Austria

**Keywords:** Meningitis C, Vaccination coverage, Prevalence

## Abstract

Current Advisory Committee on Immunization Practices (ACIP) guidelines recommend immunization of all human immunodeficiency virus (HIV)-infected patients against meningitis serotype ACWY due to recent outbreaks of meningitis C in homosexual men in the USA. Implementation of this recommendation in other countries, such as Austria is hindered by the scarce knowledge on the vaccine coverage. In this study the serostatus for meningococcus serogroup C was analyzed in 390 HIV-infected individuals residing in Austria. These individuals were representative for the Austrian HIV cohort regarding sex, age, transmission risk and HIV progression markers. Overall, 73% were on suppressive antiretroviral therapy, the mean CD4 cell count was 599 cells/μl and immunoglobulin G (IgG) seropositivity was 18% for meningococcus serogroup C. Migrants and patients who had acquired an infection via heterosexual intercourse had a higher chance for meningococcus serogroup C seropositivity. Importantly due to the well-preserved immune status of nearly all participants vaccination would be feasible in the majority of the seronegative patients. It is assumed that this measure would largely reduce the number of patients at risk for this vaccine-preventable disease.

## Introduction

Vaccines are highly effective to prevent infectious diseases in humans. Patients infected by human immunodeficiency virus (HIV) are more likely to acquire and experience a more severe course of infectious diseases and also bear a higher risk to show an inadequate response to immunization or develop immunization associated complications due to severe immune depletion [1, 2]. In times of highly efficient antiretroviral therapy immune recovery can be achieved in the majority of patients resulting in acceptable immunization rates [3]. As a consequence, HIV care guidelines recommend screening for immunization gaps and immunization of susceptible patients for an increasing number of indications [4]. Thus, in 2016 the Advisory Committee on Immunization Practices (ACIP, Centers for Disease Control and Prevention, Atlanta, GA, USA) recommended that all HIV-infected patients susceptible to meningococcal disease should be vaccinated [5]. This recommendation is based on the observation of several clustered outbreaks of invasive meningococcal disease in young men having sex with men (MSM) in several cities of the USA, Canada and Europe [6–9]. All these episodes were due to serotype C and characterized by an especially high incidence and mortality in HIV-infected MSM. In some cases, targeted immunization campaigns were able to rapidly end these outbreaks as described for Toronto or Chicago [6, 7]. Furthermore, it has been suggested that the absence of such outbreaks in the Netherlands is due to the high immunization rate in young men [10].

In Austria, the vaccination schedule does not contain any specific recommendations to immunize HIV-infected patients against any meningococcal diseases. Immunization is recommended but not reimbursed for toddlers. Reimbursement is only provided for the immunization against meningococcus serotypes A and C, W135 and Y (MEC-4) at the age of 10–13 years [11]. No catch-up programs for young adults are available and immunization rates in adults in Austria are currently not known. Up to now the general ACIP recommendation has not been taken up by any European national HIV guidelines. In order to provide a rational basis for deciding on this issue in Austria the serostatus to meningococcus C in a representative Austrian HIV cohort has been analyzed.

## Patients and methods

### Patients

This cross-sectional study was conducted at the HIV outpatient clinic of the Department of Dermatology, Medical University of Vienna. The study was approved by the local ethics committee and informed consent was obtained from every patient prior to inclusion. Adult HIV-infected patients on regular follow-up from November 2012 to February 2013 were included. Serum samples were obtained during routine check-ups. Sociodemographic and HIV-related information was collected from electronic charts.

### Sample preparation and analysis

Serum samples were prepared from blood samples by centrifugation (2000 × *g*, 10 min) and stored immediately at −20 °C. Samples were then tested for IgG antibodies against meningitis C (Alpha Diagnostics, San Antonio, TX, USA) and classified according to the manufacturer’s protocol. Samples with a value indicating adequate immune protection (>1 IU/ml) were interpreted as positive according to the manufacturer’s protocol.

### Statistics

Continuous data are presented as mean ± standard deviation (SD), categorical data as count and relative frequency. The association between seropositivity and potential predictors was assessed including demographics and HIV related factors. For univariate hypothesis testing the χ^2^-test, Fisher’s exact test and the Mann Whitney U‑test were used, as appropriate. Univariate odds ratios with 95% confidence intervals (95% CIs) were calculated for each predictor. Multivariable logistic regression models were used to assess independent predictors for seropositivity by entering these covariates stepwise into the models. For data management and analyses MS Excel 2011 for Mac and SPSS were used. Generally, a two-sided *p*‑value less than 0.05 was considered statistically significant.

## Results

In total, 390 HIV‑1 infected patients were included. The mean age was 45 years, the majority of the participants (74%) were male. The most common HIV transmission mode was that of MSM (47%), 33% acquired infection via heterosexual intercourse, and 14% had a history of (prior) intravenous drug use (IVDU). Among the 390 included patients, 144 (37%) were migrants, most of them originating either from eastern Europe/central Asia (*n* = 43, 11%), sub-Saharan Africa (*n* = 32, 8%) or resource-rich areas in western/central Europe, the USA or Australia (*n* = 47, 12%). On average HIV infection had been known for 10 years (±8 years standard deviation, SD) and 96% of the tested patients were on antiretroviral therapy (ART) for a mean of 8 years (±7 years SD). Mean CD4 value at time of blood sampling was 599 cells/µl (±296 cells/µl SD) and viral load was below the limit of quantification (<20 copies/ml) in 73% of the study cohort. Only 18 patients (5%) had a current CD4 count <200 cells/µl; however, 91% of the patients had experienced a CD4 nadir below 500 cells/µl and 47% below 200 cells/µl.

Of the 390 patients 69 (18%) tested positive for anti-meningococcal group C IgG. Neither age nor sex distribution varied between meningococcal group C IgG positive and negative patients. There was no association with the current CD4 cell count or the CD4 nadir. Duration of HIV infection and therapy was slightly shorter in IgG positive than in IgG negative individuals (Table 1).

**Table 1:** Characteristics of patients according to serostatus for meningococcus serogroup C

		MenC	
		IgG negative *n* = 321 (82%)	IgG positive *n* = 69 (18%)
Age (years, mean ± SD)		45 ± 11	45 ± 11
Male (%)		75.7	70.6
Migration background (%)		34	52
Transmission mode	MSM (%)	50.2	38.2
	Heterosexual transmission (%)	29.3	50.0
	IVDU (%)	15.1	10.3
Duration of infection (years, mean ± SD)		10.6 ± 8.0	8.8 ± 6.4
Duration of ART (years, mean ± SD)		7.7 ± 6.7	6.8 ± 5.8
CDC stage	A (%)	62.0	70.6
	B (%)	16.8	10.3
	C (%)	19.9	19.1
VL BLQ (%)		73.8	70.6
Current CD4 cell count (cells/µl, mean ± SD)		603 ± 303	584 ± 264
CD4 nadir	<500 c/µl (%)	91.0	89.9
	<200 c/µl (%)	47.7	46.4
	<50 c/µl (%)	15.6	10.1

*VL* viral load, *BLQ* below limit of quantification, *IVDU* intravenous drug use, *MSM* men who have sex with men, *ART* antiretroviral therapy, *CDC* Centers for Disesase Control and Prevention, *MenC* meningococcus serotype C, *IgG* immunoglobulin G

**Fig. 1:**
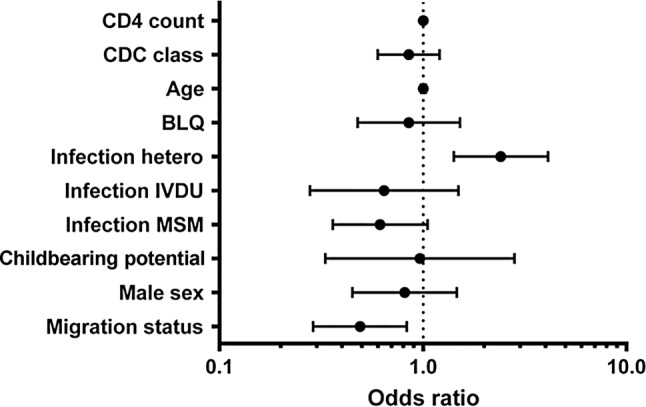
Univariate analysis of risk factors associated with meningococcus serogroup C IgG positivity. *BLQ* below limit of quantification, *IVDU* intravenous drug use, *MSM* men who have sex with men, *hetero* heterosexual

A positive correlation for IgG seropositivity against *Meningococcus* serogroup C was found for subjects with migration background, whereas subjects originating from Austria were less likely to be seropositive (odds ratio, OR 0.49, 95% confidence interval, CI 0.288–0.831, *p* = 0.007) [18, 19]. Furthermore, patients who had acquired the HIV infection via heterosexual intercourse had a higher chance to be seropositive for anti-meningococcal group C IgG (OR 2.41, 95% CI 1.413–6.106, *p* = 0.001) (Fig. 1).

In the multivariable analysis of risk factors there was no significant association between migration, sex, duration of infection, current CDC stage, CD4 count, VL or CD4 nadir and Meningococcus C IgG serostatus. The only significant association found was that between transmission route (heterosexually acquired HIV infection OR 2.28, 95% CI 1.222–4.269, *p* = 0.01) and serostatus.

## Discussion

Invasive meningococcal disease, although predominantly affecting small children, adolescents and old people, can occur at any age. Recently, outbreaks affecting young to middle-aged MSM drew attention to this disease [6–8]. A high rate of HIV coinfections and especially high mortality in this subgroup led to immunization recommendations by the ACIP [5]; however, data on seropositivity rates in European adults are scarce and to our knowledge no data on the seroprevalence of protective anti-meningococcal IgG for HIV-infected individuals in Europe are available.

Previous studies have revealed that in spite of regular and intense contact with the healthcare system immunization rates for vaccine-preventable diseases are alarmingly low in HIV-infected adult patients in Europe [12, 13]. In line with this only 18% of the patients were found to test positive for serogroup-specific IgG indicating putative seroprotection against meningococcus serogroup C. This rate is much lower than seen in children and adolescents in countries with meningococcal C immunization programs, such as the UK or the Netherlands but similar to data on adults from the general population aged 30 years and above from these countries [14, 15].

Vaccines can provide protection against five of the six major disease-causing meningococcal serogroups (A, B, C, W and Y). Introduction of these vaccines has proven effective in reducing the disease burden in several countries in the general population [16]. Furthermore, immunization also leads to reduction in the prevalence of carriage and thus indirect protection [17]. Nevertheless, individual protection seems to wane quickly, especially after early childhood immunization [18–20]. This observation probably explains the age-dependent seroprotection rates, has led to several modifications of immunization schedules and suggests the need of booster doses to maintain protection over the years. In HIV infections efficient immunization programs were hampered for a long time by the severe immune suppression that resulted in low response rates, impaired antibody titers and did not allow the use of live, attenuated vaccines. In contrast to the pre-ART era, immunization is nowadays feasible and effective in the majority of the HIV-infected population due to great improvements in the immune status. This is also seen in this study reflected by the high mean CD4 cell count. Only 5% of the patients displayed a current CD4 cell count of less than 200 cells/µl and would thus not be eligible for immunization. Durability of seroprotection is another issue when immunizing HIV-infected individuals. In light of the apparently quickly waning meningococcal serotype C protection rates in otherwise healthy individuals and the strong dependence on immunization protocols (schedule, age) applied, this issue must also be adequately addressed regarding immunization recommendations for the HIV-infected population.

In contrast to previous investigations in the same study cohort where patients with a migration background were found to show a higher need of vaccinations, in the present study migration was positively correlated with seropositivity to meningococcus serotype C [12, 13]. This might be due to a relatively high number of patients originating from high prevalence countries, including those in Africa which at least partly also belong to the so-called meningitis belt. These subjects have probably taken part in immunization programs or may have been exposed to meningococci before. In addition, HIV infection acquired via heterosexual intercourse was found to positively correlate with the serostatus, which was the only risk factor to remain in the multivariate analysis.

Taking the Austrian epidemiology into account, a strong link between heterosexual transmission mode and migration is obvious. In patients with a heterosexually acquired HIV infection the proportion of patients originating from a country other than Austria was much higher than in the MSM and IVDU risk group, especially regarding high prevalence countries [21].

We are aware that our study has several limitations. Individual vaccine and infection history has not been taken into count, furthermore data on time of migration is missing. Nonetheless we think that the data presented here are of great relevance and interest since we have included an adequate number of patients under current care, reflecting the HIV epidemiology regarding sex, age and transmission risk in Austria. Due to similarities with other (central and western) European HIV cohorts we are also quite confident that the data acquired here can be extrapolated to these populations. We thus think that our data give reliable information on seroprevalence of meningococcal C IgG in the HIV-infected population and thus could serve as a basis for immunization recommendations for HIV-infected individuals in Europe.
